# Outpatient kidney recovery after acute kidney injury requiring dialysis: a systematic review protocol

**DOI:** 10.1186/s13643-019-1134-1

**Published:** 2019-08-22

**Authors:** Carol Wang, Swapnil Hiremath, Lindsey Sikora, Manish M. Sood, Jennifer Kong, Edward Clark

**Affiliations:** 1 Department of Medicine, University of Ottawa, CPC 162 737 Parkdale Avenue, Ottawa, Ontario K1Y 1 J8 Canada; 2 Division of Nephrology, Department of Medicine, The Ottawa Hospital and University of Ottawa, The Ottawa Hospital – Riverside Campus, 1967 Riverside Drive, Ottawa, Ontario K1H 7 W9 Canada; 3 Health Sciences Library, University of Ottawa, 451 Smyth Road, Ottawa, Ontario K1H 8 M5 Canada; 4 Division of Nephrology, Ottawa Hospital Research Institute, The Ottawa Hospital, 1967 Riverside Drive, Ottawa, Ontario K1H 7 W9 Canada

**Keywords:** Acute kidney injury, Dialysis, Prognostic factors, Kidney recovery, Systematic review

## Abstract

**Background:**

Acute kidney injury requiring dialysis (AKI-D) during hospitalization is associated with both in-hospital and post-discharge mortality. Its incidence has risen over time in Canada and the USA. While the majority of AKI-D will recover to dialysis independence at the time of hospital discharge, 10–30% will transition to outpatient dialysis. The risk factors that determine dialysis independence after AKI-D and its optimal outpatient management remain unclear. Eliciting prognostic predictors of kidney recovery in patients who remain on dialysis after hospital discharge will guide subsequent clinical decision making. The objective of this study is to assess the association between patient- and treatment-related factors with short- and long-term outcomes in patients who remained dialysis-dependent after hospitalization with AKI-D.

**Methods:**

A literature search in EMBASE, MEDLINE, and PubMed will be performed based on pre-specified criteria. There are no restrictions on language and publication dates. The supplemental search will include manual scan of bibliographies of eligible studies and grey literature assessment. Pre-specified criteria will be used to select eligible studies. Relevant data will be extracted and quality assessments performed per validated tools. Qualitative data synthesis will be performed to reflect directions of associations. Meta-analysis will be formed if two or more studies with similar prognostic factors, outcomes, and adequate quality are identified. Strength of association will be quantified as odds ratios. Reporting of this review will be guided by recommendations of the Preferred Reporting Items for Systematic Reviews and Meta-Analyses (PRISMA) statement.

**Discussion:**

This systematic review aims to synthesize association between modifiable and non-modifiable prognostic factors with renal outcomes in AKI-D patients who remain dialysis-dependent after hospital discharge. Our findings will help inform the development of evidence-based management and guide long-term treatment planning for AKI-D patients.

**Systematic review registration:**

PROSPERO registration number CRD42019127394.

## Background

Acute kidney injury requiring dialysis (AKI-D) occurs in 1–2% of hospitalized patients [1, 2]. In the USA, the incidence of AKI-D has increased in the span of 2000–2014, especially in patients with diabetes [3]. A retrospective population-based study in Ontario, Canada, demonstrated a fourfold increase in AKI-D from 0.8 to 3% between 1996 and 2010 [4]. AKI-D is also associated with increased risk of both in-hospital and post-discharge mortality [1, 5]. Approximately 10–30% of patients with AKI-D who survive to hospital discharge will require outpatient dialysis [6]. Furthermore, 1 in 12 of those patients with kidney recovery to dialysis independence at the time of discharge will eventually need to resume it within 3 to 5 years’ time [1]. The factors that determine the extent of kidney recovery after AKI-D remain unclear and are likely to consist of a range of modifiable and non-modifiable factors [2].

An improved understanding of predictors of kidney recovery to dialysis independence in outpatients who require ongoing dialysis after a hospital encounter with AKI-D (as well as the gaps in knowledge in this area) can improve patient-centered decision-making and clarify the need for further research in this area. For patients unlikely to have kidney recovery, earlier and better-informed decision-making regarding long-term arteriovenous access, home therapies, and transplantation would be facilitated. This could improve patients’ quality of care; improve access, integration, and utilization of chronic kidney disease system resources; and improve access to transplant and home dialysis. For caregivers of patients more likely to recover, frequent assessment for recovery and strategies to limit further kidney damage (e.g., avoidance of intradialytic hypotension and nephrotoxins) may facilitate shorter recovery times and increase the likelihood of recovery to dialysis independence. Lastly, a better understanding of the risks of death and dialysis dependence would help patients navigate their options regarding palliative/conservative care after having gained some experience as to what is involved in undergoing maintenance dialysis in the outpatient setting.

The optimal outpatient management of AKI-D patients remains uncertain. Currently, no evidence based-guidelines exist for the best management of AKI-D patients in order to optimize kidney recovery [1, 2]. Many observational studies have reported on predictors of kidney recovery prior to hospital discharge [2, 5–7]. Nonetheless, some patients who require ongoing outpatient dialysis after hospital discharge will go on to recover kidney function to dialysis independence. Much less is known about risk factors that influence kidney recovery in the outpatient setting after a hospital encounter with AKI-D. Here, we present a protocol for a systematic review that will seek to assess the association between both patient- and treatment-related factors (modifiable and non-modifiable) with short- and long-term outcomes in patients who remained dialysis-dependent after a hospitalization with AKI-D.

## Methods

### Eligibility criteria

#### Types of studies

All studies that have reported on predictors of kidney recovery and clinically relevant outcomes (listed below) in AKI-D patients requiring post-hospitalization dialysis are eligible. These studies must present predictive/prognostic factors from baseline to follow-up period. In addition, studies must be full-text, peer-reviewed articles. This review will include both observational and interventional studies. Case reports or case series of less than or equal to 10 patients will be excluded. No restrictions will be placed on the study duration, study period, or date of publication. Studies must have analyzed potential predictors of kidney recovery in AKI-D patients who remain dialysis-dependent after hospital discharge. Only original peer-reviewed literature will be included. Studies published in all languages will be included. In addition, the following criteria must also be met:

*Patient populations*: Patients 18 years or older with acute kidney injury treated with dialysis of any type during a hospitalization who transition to outpatient dialysis after hospital discharge. Those with pre-existing end-stage renal disease defined as estimated glomerular filtration rate (eGFR) < 15 ml per minute per 1.73 m^2^ prior to hospitalization will be excluded.

*Variables of interest or predictive factors*: Any independent variable examined for potential prognostic ability will be included. We will begin with a broad study approach that spans a variety of prognostic factors which will subsequently be grouped into relevant domains. Potential domains include demographics (e.g., gender, age, ethnicity, pre-existing comorbidities), hospitalization-related factors (such as etiology of acute kidney injury, volume status at initiation of renal replacement therapy (RRT), ward versus intensive care unit admission, duration of hospitalization, urinary output at the time of hospital discharge), and treatment-related factors including modality of RRT initiated in hospital and characteristics of RRT after discharge.

#### Outcomes

The primary outcome of interest in this systematic review is kidney recovery to dialysis independence within 90 days after hospital discharge. This time interval is regarded as the threshold at which most patients would be considered to have reached end-stage renal disease [1]. The secondary outcomes of this study include kidney recovery to dialysis independence at any time point after hospital discharge, death at any time point after hospital discharge, progression to end-stage renal disease, and long-term RRT dependence, kidney transplantation, serum creatinine, and/or estimated glomerular filtration rate amongst those that recover to dialysis independence.

#### Search strategy

No prior published studies that addressed similar topics were found in PubMed, MEDLINE (via Ovid), and EMBASE (via Ovid). Similar systematic review study protocols have not been registered on PROSPERO. A health sciences librarian (LS) searched the following databases: MEDLINE and MEDLINE in Process (via OVID), Embase Classic + Embase (via OVID), Cochrane’s Central Registry for Randomized Controlled Trials, CENTRAL (via OVID), and PubMed. Appendix 1 demonstrates our search strategy, which was initially developed in MEDLINE then translated into other databases. All databases will be searched from the date of inception to April 1, 2019. No language exclusion criteria nor other publication restrictions were incorporated into the search strategy. All references will then be entered into EndNote (version X9, Clarivate Analytics Inc., Philadelphia, PA) citation manager for processing. Supplemental search will consist of manual scanning of bibliographies in eligible studies, grey literature search of clinical trial registries such as *clinicaltrials.gov*, and screening for relevant titles in the first three pages of the Google Scholar search. Relevant articles will be screened at two levels: title/abstract then full-text screening, by two reviewers (CW and EC) in the systematic review software, Covidence (Veritas Health Innovation LTD).

## Study records

### Data management

Relevant data will be extracted from Covidence (Veritas Health Innovation LTD) and managed with Microsoft Excel. Data will be synthesized with the Comprehensive Meta-Analysis (version 2, Biostat) software.

### Data collection process

Two investigators (CW and EC) will independently screen relevant study titles and abstracts per pre-specified criteria to determine their eligibilities for full-text assessment. The same investigators will subsequently examine the full texts of the selected studies according to the inclusion and exclusion criteria. Relevant studies published in English or French will be included. Disagreements will be resolved with discussion until consensus is reached. A PRISMA diagram will be generated to document the study selection process [8].

## Data extraction

A data extraction form will be created and populated with the following information:

1. Patient demographics: age, gender, ethnicity.
2. Investigated independent variables/predictors:
  Patient comorbidities: baseline creatinine (if available), pre-existing chronic kidney disease, diabetes, congestive heart failure, etc.
  Hospitalization-related factors: etiology of acute kidney injury, volume status at initiation of RRT, ward versus intensive care unit admission, duration of hospitalization, urine output at the time of hospital discharge.
  Treatment-related factors: modality of RRT initiated in hospital, characteristics of outpatient RRT after discharge.
3. Outcomes: kidney recovery after hospital discharge, mortality at any time point after hospital discharge, progression to end-stage renal disease with transition to long-term RRT, eGFR at the time of kidney recovery, time from hospital discharge to kidney recovery, and kidney transplantation.
4. Study characteristics: author, publication year, number of study participants, type of study (research design), and duration of follow-up.

## Quality assessment

The qualities of reporting and risk for bias of each included study will be appraised using validated tools. Prognostic studies that meet the inclusion criteria will be assessed for internal validity with the Quality In Prognostic Studies (QUIPS) tool [9]. The quality of reporting of observational studies will be assessed in accordance with Strengthening the Reporting of Observational Studies in Epidemiology (STROBE) checklist [10, 11]. The Cochrane Risk of Bias Assessment Tool for Non-Randomized Studies of Interventions (ROBINS-I) will be utilized to evaluate observational studies that assessed interventions [12]. The strength of the emerging evidence will be evaluated using the Grading of Recommendations Assessment, Development, and Evaluation (GRADE) method [13]. These assessments will be performed by two investigators (CW and EC) independently, and disagreements will be resolved by consensus or involvement of a third reviewer (SH) if required.

## Data synthesis

### Qualitative/narrative synthesis

A narrative synthesis will be performed to reflect directions of associations between prognostic factors and outcomes of interest. These associations will be reported as positive, negative, or none for each domain of risk factors.

### Quantitative synthesis

Meta-analyses will be performed if the data is appropriate for quantitative synthesis. Minimum of two studies is required to provide data of the same prognostic factors pertaining to outcomes of interest to permit data pooling and synthesis. The strength of association between identified prognostic factors and outcomes will be quantified as odds ratios for each outcome of interest. Cochran’s Q and the *I*^2^ test will be used to examine the statistical heterogeneity between studies [13]. Publication bias will be assessed by visual examination of the funnel plot and using the Egger test [14]. Statistical analyses will be conducted using the generic inverse variance method in Review Manager software (RevMan, version 3).

### Protocol amendments

Any amendments to the protocol will be outlined in an addendum made to specify and justify changes. These modifications and their justifications will also be included in the final report.

## Discussion

AKI-D during hospitalization is a frequent occurrence and is associated with poor outcomes. To date, there is a paucity of data on the epidemiology and outcomes of patients with AKI-D who remain dialysis-dependent at hospital discharge. Our proposed systematic review will synthesize what is known about the association of patient and treatment-related factors with short- and long-term outcomes in this population. We hope to shed light on modifiable prognostic factors that influence renal outcomes. The results could help inform the development of evidence-based clinical decision-making both during and after hospitalization of AKI-D patients in addition to guide patient counseling and navigate long-term care planning.

## Data Availability

The datasets used and/or analyzed during the current study are available from the corresponding author on reasonable request.

## Appendix 1

Ovid Technologies, Inc. Email Service Search for: 39 and 52 and 66Results: 147

Database: Ovid MEDLINE(R) and Epub Ahead of Print, In-Process & Other Non-Indexed Citations and Daily <1946 to October 09, 2018 > Search Strategy:

1 exp Acute Kidney Injury/ (41734)

2 (acute adj (kidney or renal or nephr* or tubular or dialys*)).ti. (23152)

3 (acute adj2 (kidney or renal)).tw. (45381)

4 ((crescent* or progressive or anca* or acute) and (glomerul* or nephrit*)).ti. (5627)

5 ((kidney or renal) adj isch?emi*).ti. (2199)

6 *Nephritis, Interstitial/ci [Chemically Induced] (942)

7 *Hemorrhagic Fever with Renal Syndrome/ (2316)

8 (induced adj (kidney injury or renal injury)).tw. (1469)

9 Oliguria/ (1261)

10 acute nephr*.tw. (1079)

11 (pre-renal or prerenal).tw. (1032)

12 ((arf or aki) and (renal or kidney)).tw. (12162)

13 ((nephropath* and (contrast* adj (medi* or induced or agent*))) or radiocontrast* or iodinated or crystal* or cast).mp. (463879)

14 (nephrotox* or (renal and toxi*)).ti. (9125)

15 renal tubul*.ti. (6387)

16 14 or 15 (15370)

17 ci.fs. or contrast medi*.tw. or induced.mp. (2571306)

18 16 and 17 (8116)

19 ((kidney or renal) adj isch?emi*).tw. (4807)

20 Kidney Tubules, Proximal/ (12488)

21 ur?emi*.ti. (14507)

22 renal inflammation.tw. (1127)

23 19 or 20 or 21 or 22 (32601)

24 *Reperfusion Injury/ (19063)

25 isch?emi* reperfusion.tw. or injury.ti. or acute.tw. (1248297)

26 24 or 25 (1250950)

27 23 and 26 (7078)

28 h?emolytic ur?emi*.ti. (3812)

29 (thrombotic adj (thrombocytopeni* or microangiopathy)).tw. (6588)

30 28 or 29 (9770)

31 (kidney or renal or acute).mp. (2048405)

32 30 and 31 (4803)

33 (induced adj (kidney or renal)).tw. (6968)

34 (nephrotox* or contrast medi* or reperfusion or perfusion).mp. (361366)

35 33 and 34 (2354)

36 ((tubulointerstitial or interstitial or anti-glomerular or antiglomerular) and (glomerul* or nephrit*)).ti. (3139)

37 (acute or crescentic or atypical or progressive).mp. (1509375)

38 36 and 37 (1537)

39 1 or 2 or 3 or 4 or 5 or 6 or 7 or 8 or 9 or 10 or 11 or 12 or 13 or 18 or 27 or 32 or 35 or 38 (547523)

40 exp Renal Replacement Therapy/ (192706)

41 ((kidney* or renal) adj3 replacement adj2 therap*).tw. (11268)

42 exp Renal Dialysis/ (107143)

43 ((renal or extracorporeal) adj2 dialys#s).tw. (2899)

44 exp Hemofiltration/ (6454)

45 h?mofiltration*.tw. (3280)

46 h?modialys#s.tw. (57340)

47 h?modialfiltration*.tw. (19)

48 Dialysis/ (12478)

49 cavf.tw. (37)

50 sustained low-efficiency dialysis.tw. (112)

51 sled.tw. (931)

52 or/40-51 (226540)

53 Outpatients/ (13850)

54 Ambulatory Care Facilities/ (17216)

55 outpatient clinics, hospital/ (15294)

56 Surgicenters/ (1922)

57 exp General Practice/ (72426)

58 Ambulatory Care Facilities/ (17216)

59 (ambulatory adj3 care).tw. (11644)

60 (ambulatory adj2 health*).tw. (1050)

61 (surgicent* or surgi-cent*).tw. (104)

62 ((general or family) adj2 practice*).tw. (51778)

63 (outpatient* or out-patient*).tw. (163496)

64 (posthospitalization or posthospitalisation or post-hospitalization or post-hospitalisation).tw. (752)

65 (postdischarg* or post discharg*).tw. (7564)

66 or/53-65 (301279)

67 39 and 52 and 66 (147)

## Abbreviations

AKI-D: Acute kidney injury requiring dialysis
eGFR: Estimated glomerular filtration rate
EMBASE: Excerpta Medica database
GRADE: Grading of Recommendations Assessment, Development and Evaluation
MEDLINE: Medical Literature Analysis and Retrieval System Online
PRISMA: Preferred Reporting Items for Systematic Reviews and Meta-analyses
PROSPERO: International Prospective Register of Systematic Reviews
QUIPS: Quality In Prognostic Studies
ROBINS-I: Risk of Bias In Non-randomized Studies of Interventions
RRT: Renal replacement therapy
STROBE: Strengthening the Reporting of Observational Studies in Epidemiology
